# Biogenesis of omegasomes and autophagosomes in mammalian autophagy

**DOI:** 10.1042/BST20240015

**Published:** 2024-10-11

**Authors:** Puck N. Norell, Daniele Campisi, Jagan Mohan, Thomas Wollert

**Affiliations:** Membrane Biochemistry and Transport, Institut Pasteur, Université de Paris, UMR3691 CNRS, 75015 Paris, France

**Keywords:** autophagy, nonselective autophagy, omegasome, phagophore

## Abstract

Autophagy is a highly conserved catabolic pathway that maintains cellular homeostasis by promoting the degradation of damaged or superfluous cytoplasmic material. A hallmark of autophagy is the generation of membrane cisternae that sequester autophagic cargo. Expansion of these structures allows cargo to be engulfed in a highly selective and exclusive manner. Cytotoxic stress or starvation induces the formation of autophagosomes that sequester bulk cytoplasm instead of selected cargo. This rather nonselective pathway is essential for maintaining vital cellular functions during adverse conditions and is thus a major stress response pathway. Both selective and nonselective autophagy rely on the same molecular machinery. However, due to the different nature of cargo to be sequestered, the involved molecular mechanisms are fundamentally different. Although intense research over the past decades has advanced our understanding of autophagy, fundamental questions remain to be addressed. This review will focus on molecular principles and open questions regarding the formation of omegasomes and phagophores in nonselective mammalian autophagy.

## Initiation of autophagy

**Figure 1.**
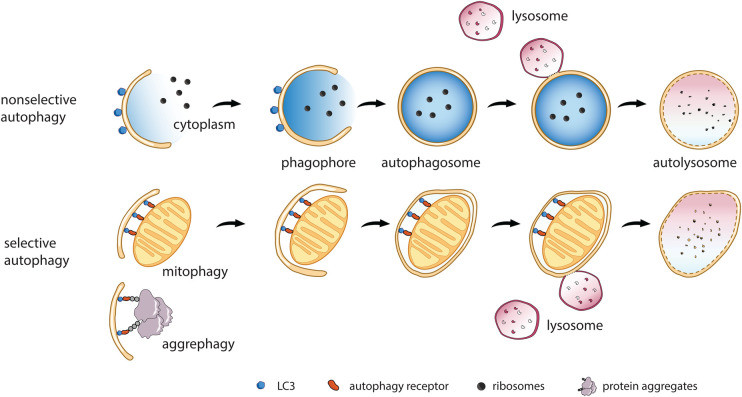
Selective and nonselective autophagy are distinct pathways. Cellular stress and starvation induce the formation of nonselective autophagosomes that mainly sequester bulk cytoplasm and ribosomes. The pathway ensures cellular survival under these adverse conditions and requires the formation of a free standing phagophore that expands in the absence of cargo. Cellular homeostasis is maintained by selective autophagy, that sequesters damaged or superfluous cellular material in double-membrane-bounded autophagosomes and delivers them to lysosomes for degradation. Autophagy receptors bind cargo and initiate autophagy and tether cargo to phagophore membranes by interacting with lipidated LC3. Both pathways rely on the autophagy core machinery, but their mode of action differs to meet different physical requirements of phagophores. Note that LC3 proteins are found on both faces of phagophores in selective and nonselective autophagy. The figure highlights one of the principal functions of LC3 proteins as scaffold component (nonselective) and cargo adaptor (selective autophagy).

Eukaryotic cells possess a highly efficient and adaptive mechanism to degrade unwanted or damaged cellular components, termed autophagy [1]. The biogenesis of autophagosomes starts with the formation of membrane cisternae, called phagophores, which sequester cytoplasmic material, enclose it, and transport it to lysosomes for degradation. In selective autophagy, the formation of phagophores is initiated by cargo, which serves as a platform for phagophore formation and expansion [2,3]. Consequently, cargo defines the size and shape of selective phagophores (Figure 1), requiring phagophore membranes to be flexible enough to engulf cargo by a zipper-like mechanism, while excluding cytoplasm and other cellular structures. In contrast, nonselective phagophores are free-standing membranes that engulf bulk cytoplasm. Because these phagophores lack structural support from cargo, their expansion needs to be co-ordinated by other mechanisms to ensure that a perfectly spherical vesicle, the autophagosome, can be formed (Figure 1). Therefore, nonselective phagophores need to possess entirely different physical properties compared with selective phagophores, being rigid enough to allow cytoplasm to be engulfed [4].

In mammalian cells, nonselective autophagy is initiated at specific domains of the endoplasmic reticulum (ER) called omegasomes, whose shape resembles the Greek letter omega (Ω) [5]. Omegasomes can adopt cisternae-like or vesicular-tubular morphologies, suggesting that they possess structural plasticity [6–8]. Morphological studies of omegasomes using electron microscopy suggest that their membranes are continuous with the ER [5], a view that has recently been challenged by *in situ* cryo-electron tomography (cryo-ET) [7]. Omegasomes are enriched in PtdIns3P, and their major purpose is to promote the formation of cup-shaped phagophores by acting as platforms for phagophore nucleation [6].

Omegasome formation starts with the recruitment of the ULK1 complex (comprising ULK1/2 kinases, FIP200, ATG13, and ATG101) and PI3KC (comprising Beclin1, ATG14L, VPS34, and VPS15) to the ER. The process is induced by inactivation of mTORC1 in response to amino acid starvation, or by activation of AMP-activated protein kinase (AMPK) due to glucose starvation [9]. In fed conditions, the mTORC1 complex phosphorylates serine residues in ATG13 and ULK1/2, suppressing the kinase activity of ULK1 and the recruitment of the ULK1 complex to the ER [10,11]. Starvation inactivates mTORC1, and the rapid dephosphorylation of ATG13 and ULK1/2 initiates autophagy. AMPK phosphorylates different residues in ULK1, thereby stimulating its kinase activity to promote autophagy [12]. The recruitment of ULK1 and PI3KC complexes enriches the ER in PtdIns3P and allows effector proteins, such as the canonical omegasome marker DFCP1 to be recruited [5,13]. The colocalization of ULK1 and PI3KC at the ER is accompanied by the recruitment of ATG9 vesicles which are thought to nucleate phagophores [14–16].

Although the autophagic machinery is highly conserved from yeast to humans, the molecular mechanisms of autophagy differ significantly. In yeast cells, phagophores do not emerge from the ER, but are assembled at specific sites of the vacuole at which clustering of the tethering protein Vac8 leads to the recruitment of the PI3K and the Atg1 kinase complexes [17,18]. Atg9 vesicles are recruited by Atg17 [19,20], which is a yeast-specific subunit of the Atg1 kinase complex. Tethering of Atg9 vesicles by Atg17 and subsequent fusion generates the phagophore precursor [21,22]. Furthermore, clustering of the Atg1 kinase complex by Vac8 activates the kinase activity of Atg1 to initiate autophagy [23].

## Dynamics and lipid sources

**Figure 2.**
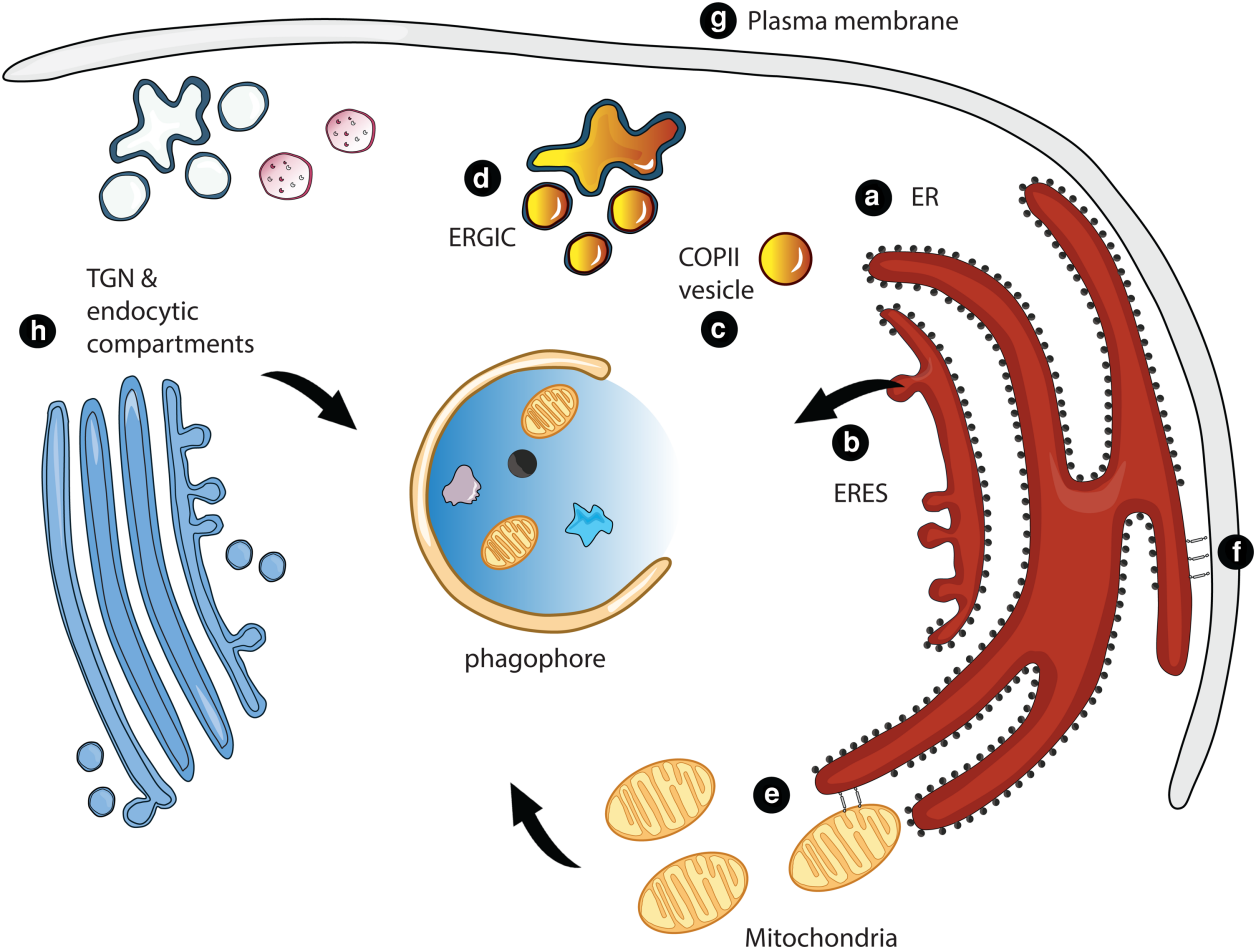
Membrane sources in nonselective autophagy. Several organelles provide lipids for the nucleation and expansion of nonselective autophagosomes, including the endoplasmic reticulum (ER, **a**), ER-exit sites (ERES, **b**), COPII vesicles (**c**), the ER-Golgi intermediate compartment (ERGIC, **d**), ER-mitochondria contact sites (**e**), ER-plasma membrane contact sites (**f**), the plasma membrane (**g**), and the Trans-Golgi Network (TGN) as well as endocytic compartments (**h**).

The formation of omegasomes occurs within 3 min, and autophagosomes are formed within 10 min [5,24,25]. The transient nature of autophagosome precursors makes it difficult to study the formation of autophagosomes and the relationship between the ER, omegasomes, and phagophores. Interestingly, a small number of omegasomes give rise to a large number of autophagosomes, suggesting that several phagophores emerge from one omegasome [24]. The vast majority of cellular lipids are generated by the ER, suggesting that the ER is a principal source of lipids for the generation of autophagosomes [26]. However, several other sources, including recycling endosomes [27,28], the plasma membrane (PM) [29,30], the trans-Golgi network (TGN) [14], mitochondria [31], the ER–Golgi intermediate compartment (ERGIC) [32,33], and ER exit sites (ERES) [34], also provide lipids for the biogenesis of phagophores (Figure 2). Recent studies have revealed that different vesicle populations merge to generate pre-autophagic structures, suggesting that vesicles provide, at least in part, lipids for phagophore expansion [35]. How such vesicles are generated, from which organelles they emerge, and whether certain cellular conditions induce the mobilization of lipids from distinct sources remain central questions in autophagy research.

**Figure 3.**
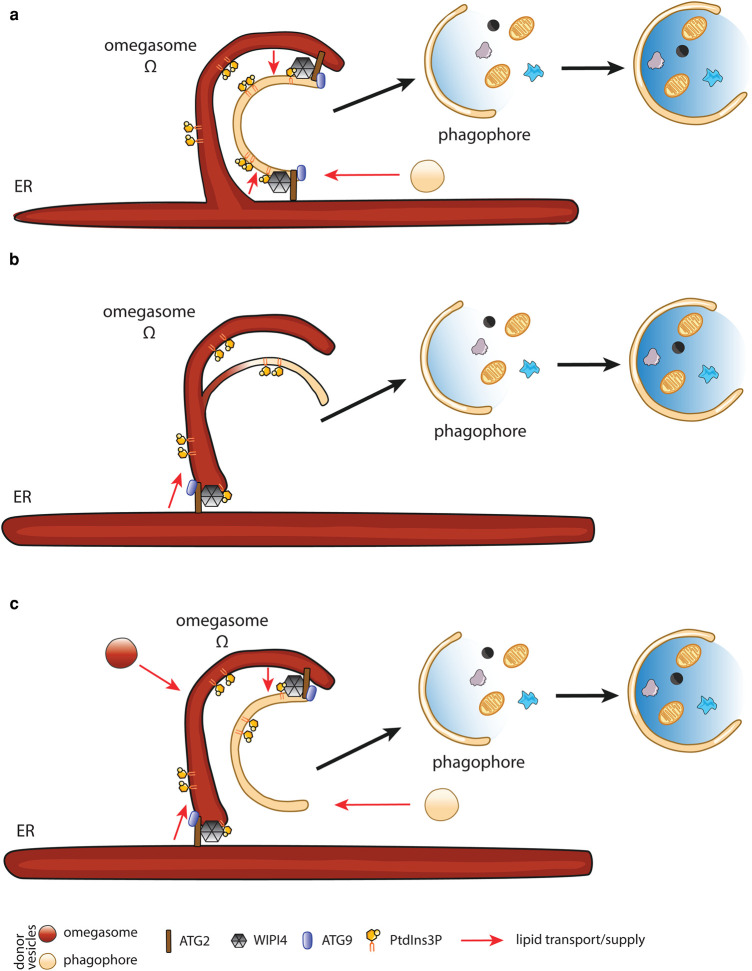
Omegasomes and their relationship to ER and phagophores. Omegasomes are platforms from which autophagosomes emerge in nonselective autophagy. (**a**) Previous studies reported that omegasomes are domains of the ER that act as platforms for the *de novo* formation of autophagosomes. Lipids required for the nucleation and expansion of phagophores are provided by vesicles or through direct lipid transfer from the ER and/or omegasomes by ATG2. (**b**) Recent studies suggested that omegasomes are not connected to the ER, indicating that they are cleaved off the ER after their formation. Phagophores may emerge by a budding mechanism through remodeling of omegasomal membranes. The ER can donate lipids by ATG2-mediated direct transfer. (**c**) Omegasomes might also form by vesicular fusion and through lipid transfer from the ER. Phagophore formation might occur by vesicular and ATG2 mediated lipid transfer. However, phagophores might also be derived from omegasomes (as shown in **b**). In this case, lipids would be delivered to omegasomes and used for the production of phagophores by remodeling.

Contact sites between organelles allow the dynamic exchange of lipids. Phagophores form such contacts with the ER (Figure 3a). Recent studies have revealed that ER-phagophore contact sites are established by the lipid transporter ATG2 [36–38]. ATG2 is a 16 nm-long tethering protein that possesses a central hydrophobic channel [39]. Its N-terminal Chorein domain binds the ER-resident lipid scramblases TMEM41B and VMP1 [40], while its C-terminus interacts with the phagophore-resident scramblase ATG9 [41]. Moreover, ATG2 forms a complex with the PtdIns3P-binding protein WIPI4, which supports targeting of ATG2 to PtdIns3P-enriched membranes of the phagophore [42,43]. This configuration suggests that ATG2 transports lipids between the ER and phagophores while the scramblases ensure an equal distribution of lipids between the two membrane leaflets. Recent *in vitro* reconstitution experiments demonstrated that ATG2 indeed transfers lipids between vesicles with an estimated transport rate of between 0.017 per second (s^−1^) and 115 s^−1^ [44,45].

Generating autophagosomes with a diameter of ∼400 nm requires ∼3 million lipids, which would require ∼90 ATG2 molecules to provide enough lipids within 10 min [44]. This suggests that direct lipid transport by ATG2 is supplemented by other lipid transport mechanisms, including vesicular transport. Moreover, the nature of the ER membrane, being heavily populated by integral and peripheral membrane proteins, needs to be considered. More than 50% of the mass of ER membranes is composed of proteins [46]. Phospholipids that are in direct contact with transmembrane proteins are tightly bound, suggesting that a significant number of phospholipids are sequestered and cannot be extracted from the membrane. Consequently, these lipids are not available for any transport mechanisms. However, a potential lack of ER-resident lipids for phagophore expansion can be offset by an ‘on-demand’ synthesis. In fact, recent studies have revealed that Faa1 colocalizes with phagophores, channels activated fatty acids to phospholipid synthesis, and co-ordinates integration of phospholipids into phagophore membranes [47].

Taken together, three major lipid transport modalities appear to contribute lipids for autophagy, including vesicular transport, direct lipid transfer, and *de novo* lipid synthesis. The challenge for future studies will be to determine how many lipids are contributed by the different pathways and whether the flow of lipids through these pathways can be dynamically modulated in response to cellular and environmental cues.

## The role of the ER in autophagosome biogenesis

The most prominent domain of the ER from which nonselective autophagosomes emerge was discovered by Axe and colleagues more than 16 years ago [5]. Because of the characteristic shape of these peculiar ER domains, they have been called omegasomes (Figure 3). Despite intense research over the past decade, the molecular mechanism by which omegasomes are formed remains unknown. Their formation is initiated by the recruitment of the PI3KC and the ULK1 kinase complexes to the ER. The phosphorylation of PtdIns by PI3KC is a key step in the formation of omegasomes and results in the recruitment of DFCP1. A recent study revealed that DFCP1 is an ATPase that constricts omegasomes to release autophagosomes, suggesting that autophagosomes are derived by extrusion from the omegasome [48]. Moreover, DFCP1 regulates the biogenesis of lipid droplets [49], which in turn are involved in the formation of autophagosomes [50], implying that under certain conditions, lipid droplets can nurture phagophore expansion in a process co-ordinated by DFCP1.

Previous studies reported that omegasomes are continuous with the ER [8,51], suggesting that the formation of omegasomes occurs by budding from the ER membrane (Figure 3a). More recent developments in cryo-ET allow cellular structures to be preserved by cryo-fixation instead of classical methods, which involve chemical fixation and dehydration of samples. Using these techniques, contact sites between omegasome-like membrane cisternae and the ER have been frequently observed, while no continuous membrane bridges were identified, suggesting that omegasomes and phagophores are distinct entities that are not continuous with the ER [7,52,53] (Figure 3b,c). However, these contact sites were frequently populated by stick-like densities, and the length of these structures was in agreement with the length of ATG2, suggesting that ATG2 stabilizes contact sites between the ER and omegasomes (Figure 3b,c). Given that ATG2 is a lipid transporter, this observation also suggests that direct lipid transfer from the ER promotes the biogenesis of omegasomes.

The lack of membrane bridges between the ER and omegasomes also suggests that if omegasomes are subdomains of the ER, they need to be cleaved off the ER after they have been formed. Moreover, omegasomes and the ER have very distinct physical characteristics. The luminal distance between the inner and outer membrane of the omegasome is ∼23 nm, which is much thinner than that of ER sheets or tubes. The intermembrane space of omegasomes is devoid of protein content, and canonical ER proteins do not colocalize with omegasomes [7]. Thus, if omegasomes are derived by remodeling of the ER, a yet to be identified highly specialized remodeling machinery would be required.

Another possibility is that omegasomes are nucleated by vesicular fusion and expanded by vesicular and ATG2-mediated lipid supply (Figure 3c). This possibility is supported by recent data showing that ATG16L1 and FIP200 vesicles undergo heterotypic fusion to form the HyPAS [35]. Interestingly, FIP200 vesicles are derived from the Golgi, while ATG16L1 vesicles emerge from recycling endosomes. In support of this scenario, earlier studies reported that ATG16L1 and ATG9 vesicles merge at recycling endosomes, and RAB11A-positive recycling endosomes serve as platforms for phagophore formation [27]. Whether the HyPAS or RAB11A-positive endosomes are related to omegasomes remains unclear, and further studies are needed to establish this relationship.

Not only the origin of omegasomes but also their relationship to phagophores remain controversial. The concept that omegasomes are platforms for the generation of phagophores was closely associated with the notion that phagophores are formed *de novo* by vesicular fusion involving vesicular carriers of various origins [15,27,29,33,54]. Recent *in vivo* cryo-ET studies characterized the morphology of phagophore rims and reported that they are in contact with omegasome-like structures distinct from ER cisternae (Figure 3c) [7]. Moreover, phagophore rims appear dilated and contain granular electron densities, which is not the case for rims of omegasome-like structures [7,52,55], reinforcing the concept that omegasomes and phagophores are distinct, separate structures (Figure 3a,c).

Moreover, expanding phagophores are often associated with ER membranes, forming contact sites where autophagy regulators are recruited [56,57]. Notably, the lipid scramblase ATG9 and the lipid transfer complex comprising WIPI4 and ATG2 stabilize these contact sites [37,39,43], suggesting that the expansion of phagophores involves direct lipid transfer from the ER (Figure 3a).

Taken together, the ER, omegasomes and phagophores appear to be separate and distinct structures. Both omegasomes and phagophores are connected to each other and to the ER by stick-like densities, suggesting that ATG2 stabilizes these contact sites and facilitates lipid transport between these structures. Moreover, experimental data support the notion that the nucleation of omegasomes and/or phagophores involves vesicular fusion (Figure 3c). The major difficulty in untangling the exact relationship between the ER, omegasomes, and phagophores remains their dynamic nature and close proximity, making it difficult to assign experimental data to the biogenesis of omegasomes or phagophores.

## ERES and ERGIC

ERESs are specialized regions of the ER where COPII vesicles form. There is mounting evidence that ERESs are important for autophagosome biogenesis (Figure 2b). ERESs may serve as structural platforms for the generation of autophagosomes by promoting the formation of a COPII-derived compartment that recruits Atg9 and the PI3KC in yeast [34].

COPII vesicles are essential for transporting newly synthesized proteins from the ER to the Golgi, where proteins are sorted to their final destinations and most glycosylation reactions occur [55]. Several studies reported that COPII vesicles contribute to autophagosome biogenesis by delivering lipids to the expanding phagophore in *Saccharomyces cerevisiae* (Figure 2c) [33,54,58]. Trafficking of COPII vesicles to phagophores is co-ordinated by the membrane tethering complex TRAPPII and the small GTPase Ypt1 [34]. In mammalian cells, the ERGIC provides precursor membranes for the generation of autophagosomes (Figure 2d) [32,33]. Moreover, special contact sites between the ERGIC and ERESs promote the formation of COPII vesicles that act as phagophore precursors [59]. Functional ERESs are maintained by the autophagy protein LC3C, which is conjugated to ER membranes and recruits TECPR2, which in turn promotes COPII vesicle formation through its interaction with SEC24D. Mutations affecting TECPR2 function reduce COPII vesicle formation and inhibit autophagy, suggesting that the cooperation of LC3C and TECPR2 initiates autophagy by stabilizing ERESs [60]. Moreover, the recruitment of FIP200 to ERESs by SEC12 induces remodeling of ERESs, from which COPII vesicles emerge that are redirected to phagophores to promote autophagy [61]. Other autophagy-promoting COPII vesicles are derived from the ERGIC. They contain a specific combination of COPII components, including phosphorylated Sec23B and Sec24A/B [62]. The formation of specialized populations of COPII vesicles during starvation allows cells to promote autophagy, while the budding of canonical COPII vesicles from conventional ERESs for protein secretion is suppressed.

## The role of ER contact sites in autophagy

Contact sites between the ER and mitochondria (ERMCSs) are essential for mitochondrial homeostasis by transferring phospholipids from the ER to mitochondria [63]. ERMCSs are also important signaling platforms, regulating the activity of the tricarboxylic acid cycle and the oxidation of fatty acids [64,65]. Furthermore, ERMCSs serve as platforms for autophagosome biogenesis and provide lipids for phagophore expansion (Figure 2e). Depletion of the ERMCSs components MFN2 and voltage-dependent anion channel 1 inhibits autophagy, and activation of autophagy by starvation leads to the recruitment of ATG14, a component of PI3KC, and of ATG5 to ERMCSs [66,67]. ATG5 is a component of the E3-ligase complex that conjugates LC3 to the phospholipid phosphatidylethanolamine to promote autophagosome biogenesis. Mechanistically, starvation induces the dissociation of AMBRA1 from the anti-apoptotic mitochondrial protein Bcl-2, allowing AMBRA1 to interact with Beclin1 to initiate autophagy by activating PI3KC [68]. Moreover, MFN2, an essential structural component of ERMCSs, interacts with AMPK [66]. Upon disruption of the calcium flux from the ER to mitochondria at ERMCSs, the mitochondrial pool of AMPK is activated, and AMPK phosphorylates and activates Beclin1 to initiate autophagy [69]. Another study reported that the mitochondrial outer membrane protein Miga recruits ATG14 to ERMCSs to promote the phosphorylation of PtdIns by activating PI3KC [70]. Furthermore, the TOMM40/70 complex recruits ATG2A and ATG9 to ERMCSs [71], suggesting that ERMCSs promote phagophore expansion through direct lipid supply.

Apart from mitochondria, the ER is also dynamically connected with the PM. ER-PM contact sites serve as platforms for the formation of autophagosomes [72,73], and the PM provides lipids for phagophore initiation and expansion (Figure 2f) [30]. ER-PM contact sites are mediated by tethering proteins such as E-Syt2 [74]. E-Syt2 interacts with the ER-resident protein VMP1, which promotes autophagy by interacting with the PI3KC subunit Beclin1 [75]. The recruitment of Beclin1 to ER-PM contact sites induces the formation of omegasomes, to which the PtdIns3P-binding proteins DFCP1 and WIPI2 are recruited [76]. Furthermore, PM-localized ATG16L1 is involved in the formation of pre-autophagic structures [30], suggesting that the expansion of phagophores at ER-PM contact sites is supported by lipids from the PM (Figure 2g). Another population of autophagic precursors, positive for ATG9A, can also form from the PM. The two populations of PM-derived autophagy precursors coalesce at recycling endosomes, which in turn promote autophagy by acting as platforms for the biogenesis of autophagosomes [77].

## Trans-Golgi and endocytic compartments

The Golgi is the central sorting station for proteins in the secretory pathway. Furthermore, most glycosylation reactions occur in the Golgi and they are essential for producing mature glycoproteins and glycolipids. The Golgi, and in particular the TGN, promote autophagy by supplying lipids for the nucleation and expansion of autophagosomes (Figure 2h) [78]. The TGN also harbors the lipid scramblase ATG9, which promotes autophagy by providing seeds for the nucleation of phagophores [16]. Upon autophagy induction by starvation, ATG9 exits the Golgi and is targeted to the ER where it co-operates with the ULK1 and the PI3KC complexes to produce omegasomes [79,80]. ATG9 has a very complex trafficking itinerary. It shuttles through endocytic compartments, including recycling endosomes and the PM, on its way to the phagophore assembly site [14,81,82]. Despite its essential function, ATG9 is not detected in phagophore membranes, suggesting that only a few molecules are incorporated [16].

## Phagophore formation by LC3B

The recruitment of LC3B to phagophores is a hallmark of nonselective autophagy, and its covalent attachment to phagophore lipids has been widely used to identify phagophore membranes and autophagosomes [83]. The conjugation of LC3B is mediated by a ubiquitin-like conjugation machinery, including the E1-like enzyme ATG7, the E2-like enzyme ATG3, and the E3-like ligase complex comprising the ATG12-ATG5 conjugate and ATG16L1 [84].

The conjugation of LC3B to phagophore membranes promotes phagophore expansion [85,86], and depletion of LC3B and its human paralogs results in the formation of smaller autophagosomes and a maturation defect [87]. Mechanistically, lipidated LC3B assembles with its E3-ligase complex into a membrane scaffold [88]. A similar activity has previously been described for the yeast LC3B homolog Atg8 [89,90]. Importantly, our recent *in vitro* reconstitutions demonstrated that the LC3B/ATG12-ATG5-ATG16L1 scaffold shapes model membranes into membrane cups that closely resemble phagophores in nonselective autophagy [88]. Interestingly, the formation of this scaffold also explains why the localization of ATG16L1 and ATG5 is restricted to the outer face of phagophores in mammalian cells [91,92]. The remodeling reaction required an intact coiled-coil region of ATG16L1, as well as its C-terminal unstructured region, while the C-terminal WD domain was dispensable [88]. Interestingly, previous studies reported several lipid binding sites in the N-terminal domain, the coiled-coil domain, and the C-terminal unstructured region of ATG16L1 [93,94]. These membrane-binding regions are indispensable for membrane remodeling by ATG16L1 *in vitro*. We also recently reported that cup-like membranes emerged from extended ER-PM contact sites *in vivo* [88] when ATG16L1 was directed to the PM by expressing WIPI2-CAAX [95]. This observation is significant because it implies that phagophores are formed by remodeling omegasome membranes. In this model (Figure 3b), omegasomes would not only be the platform for phagophore formation but also the primary lipid source. Phagophore and omegasome membranes would thus have the same origin, and lipids for autophagosome biogenesis would primarily be supplied to omegasomes, for example by ATG2-mediated transfer from the ER, vesicular transport, *or de novo* phospholipid synthesis. The challenging future task will be to determine how these three lipid delivery modalities are co-ordinated, whether their contribution is regulated by environmental or cellular cues, and whether lipids are mainly supplied to omegasomes, to phagophores, or to both.

## Open Access

Open access for this article was enabled by the participation of Institut Pasteur in an all-inclusive *Read & Publish* agreement with Portland Press and the Biochemical Society under a transformative agreement with Individual.

## Perspectives

- Nonselective autophagy is a fundamental and highly conserved recycling pathway that allows cells to respond to cellular stress.
- Nonselective autophagosomes emerge from omegasomes, which are thought to be domains of the ER.
- Revealing the functional and structural relationship between omegasomes, phagophores and the ER remains to be addressed.

## Abbreviations

AMBRA1: activating molecule in Beclin1-regulated autophagy protein 1
AMPK: AMP-activated protein kinase
ATG: autophagy-related
Bcl-2: B-cell lymphoma 2 protein
PI3KC: class III PtdIns3-kinase complex
COPII: coat protein complex II
cryo-ET: cryo-electron tomography
DFCP1: double FYVE containing protein 1
ER: endoplasmic reticulum
ERGIC: ER-Golgi intermediate compartment
ERMCS: ER-mitochondria contact site
E-Syt2: extended synaptotagmin 2
FIP200: FAK family kinase-interacting protein of 200 kDa
HyPAS: hybrid pre-autophagic structures
mTORC1: mammalian target of rapamycin kinase complex 1
LC3: microtubule-associated protein 1A/1B-light chain 3
MFN2: Mitofusin 2
PtdIns: phosphatidylinositol
PM: plasma membrane
RAB11A: Ras-related protein 11A
TECPR2: Tectonin beta-propeller repeat-containing protein 2
TGN: trans-Golgi network
TMEM41B: transmembrane protein 41B
TRAPPII: transport protein particle II
ULK: Unc-51-like kinase
VPS: vacuolar sorting protein
VMP1: vacuole membrane protein 1
WIPI4: WD repeat domain PtdIns Interacting protein 4
Ypt1: yeast protein transport 1
